# Fascin-Centred Invasive Competence in Eutopic Endometrium: A Hypothesis-Driven Narrative Review of Endometriosis Pathogenesis and Non-Surgical Biomarker Potential

**DOI:** 10.3390/ijms27167234

**Published:** 2026-08-13

**Authors:** María Pilar Marín-Sánchez, Daimaris Ortega-Suárez, Álvaro Federico López-Soto, Iryna Kozak, Rebeca Benito-Villena, Marina Vives-Ramírez, Fátima Postigo-Corrales, Alejandra Isaac-Montero, Pablo Conesa-Zamora, Ginés Luengo-Gil

**Affiliations:** 1 Gynaecology and Obstetrics Department, Instituto Murciano de Investigación Biosanitaria (IMIB), Hospital General Universitario Santa Lucía, 30202 Cartagena, Spain; daiortsua@gmail.com (D.O.-S.); alvarolopezsoto1@gmail.com (Á.F.L.-S.); rebe.benito.villena@gmail.com (R.B.-V.); 2 Group of Molecular Pathology and Pharmacogenetics, Pathology and Clinical Analysis Departments, Instituto Murciano de Investigación Biosanitaria (IMIB), Hospital General Universitario Santa Lucía, 30202 Cartagena, Spainpconesa@ucam.edu (P.C.-Z.); 3 Health Sciences Faculty, Universidad Católica de Murcia (UCAM), 30107 Guadalupe, Spain

**Keywords:** endometriosis, fascin, FSCN1, eutopic endometrium, invasive competence, cytoskeletal remodelling, cell composition confounding, single-cell transcriptomics, menstrual effluent, non-invasive biomarkers

## Abstract

Endometriosis is a chronic, oestrogen-responsive inflammatory disease characterised by endometrial-like tissue outside the uterine cavity. Because retrograde menstruation is common, lesion establishment probably requires cellular competence and a permissive ectopic microenvironment. This hypothesis-driven narrative review evaluates fascin (*FSCN1*) as a candidate cytoskeletal effector and considers antecedent eutopic priming versus induction after ectopic adhesion. Functional evidence was integrated with a targeted public-data screen. Donor-level reanalysis of GSE179640 found no conclusive overall eutopic case–control difference and predominantly non-epithelial expression. Exploratory analysis of GSE203191 suggested higher *FSCN1* expression within a *HSPA6*+ stromal subcluster in diagnosed cases, without a comparable epithelial signal or detectable increase in subcluster abundance. This small post hoc analysis remains hypothesis-generating. *FSCN1* was absent from the published HECA stromal/macrophage differential-expression lists and was not prioritised by the 2023 endometriosis GWAS. The current evidence therefore argues against uniform epithelial or whole-eutopic overexpression but permits a lineage-restricted stromal state. Fascin participates in autophagy- and miR-145-sensitive invasion networks, although these pathways are pleiotropic. Validation requires cycle- and lineage-resolved tissue mapping, compositional controls, matched lesions, and direct *FSCN1* perturbation. Fascin should currently be regarded as a candidate multi-marker component and preclinical target, not a validated biomarker or systemic therapeutic target.

## 1. Introduction

Endometriosis is a chronic, oestrogen-responsive inflammatory disease characterised by endometrial-like glands and stroma outside the uterine cavity [1,2,3,4,5].

Although retrograde menstruation is common, only a subset of individuals develops persistent ectopic lesions. Lesion establishment is therefore unlikely to depend on exposure alone: refluxed fragments must adhere, survive, remodel actin, invade local barriers, and tolerate inflammatory and metabolic stress [4,6,7,8]. The eutopic endometrium in endometriosis has been associated with progesterone resistance, oestrogen-dominant signalling, inflammatory dysregulation, and reduced receptivity, but these changes are heterogeneous and do not establish a universal pre-invasive state [7,9,10,11,12,13,14,15].

We focus on fascin as a candidate downstream effector of these processes. The central question is deliberately bidirectional: does a rare, cell type-specific fascin-high state precede implantation, or is fascin induced mainly after ectopic adhesion by inflammation, hypoxia, oxidative stress, and other niche cues? The strongest direct evidence currently comes from experimental endometriosis systems rather than phase-controlled human eutopic tissue [16,17,18].

Accordingly, this article is a hypothesis-driven narrative review, not a demonstration of causality. We distinguish direct endometriosis evidence, indirect mechanistic analogy, exploratory public-data observations, and experimentally falsifiable predictions.

## 2. Review Methods and Public-Data Screen

Targeted searches were conducted in PubMed/MEDLINE, publisher databases, and reference lists from database inception to 20 July 2026. Search concepts combined endometriosis, eutopic endometrium, ectopic lesion, menstrual effluent, or saliva with fascin, *FSCN1*, miR-145, *PCAT1*, actin remodelling, single-cell, transcriptomics, biomarker, or invasion. English-language human studies, endometriosis-relevant experimental studies, guidelines, and authoritative reviews were prioritised. Cancer or adenomyosis studies were included only when they informed an explicitly labelled mechanistic analogy. Because the objective was conceptual synthesis rather than exhaustive effect estimation, no meta-analysis or formal risk-of-bias score was performed.

For the public-data screen, raw counts from GSE179640 [13] were analysed at donor level in control endometrium and eutopic endometrium from patients with endometriosis. Although the source dataset also contains peritoneal and ovarian ectopic lesions, these tissues were excluded from the eutopic case–control comparison reported here. The single-cell analysis comprised 3 control and 9 endometriosis donors. Broad epithelial, stromal/fibroblast, endothelial, myeloid/antigen-presenting, lymphoid, and smooth-muscle/perivascular compartments were assigned using canonical marker scores, followed by *FSCN1* detection and pseudobulk aggregation per donor. An independent bulk comparison comprised 5 control and 7 endometriosis eutopic samples. Mann–Whitney tests are reported descriptively because of the small and unbalanced groups.

To assess anatomical site effects in the complete GSE179640 single-cell dataset, *FSCN1* expression was summarised per biological sample within each original Seurat cluster. Samples containing fewer than 20 cells in a given cluster were excluded. The proportion of *FSCN1*-positive cells was evaluated using beta-binomial generalised linear mixed models, whereas log1p-transformed sample-level mean expression was analysed using linear mixed-effects models. Tissue type was included as a fixed effect and patient identity as a random intercept. Global *p* values were adjusted across clusters using the Benjamini–Hochberg procedure, followed, where appropriate, by Tukey-adjusted pairwise comparisons.

To investigate whether a more restricted stromal signal was present in an independent eutopic dataset, the processed single-cell object from GSE203191 [19] was also interrogated. The original study comprised menstrual effluent-derived endometrial tissue from 9 controls, 13 symptomatic participants without surgical confirmation of endometriosis, and 11 participants with surgically confirmed disease. For the stromal *FSCN1* analysis, only the tissue-enriched menstrual effluent preparation (“ME-tissue”) was included. The original major-cell and stromal subcluster annotations were retained, and no new reclustering or label transfer was performed. Five stromal subclusters were examined: *IL11*+, *SRGN*+, *IGFBP1*+, *HSPA6*+, and *MGP*+. One diagnosed donor lacking stromal subcluster annotations was excluded. *FSCN1* expression was summarised at subject level, and subjects with fewer than 10 cells in a given subcluster were excluded from that comparison. The *HSPA6*+ analysis included 4 controls, 7 symptomatic participants, and 5 diagnosed cases. Group differences were evaluated using the Kruskal–Wallis test, followed by Dunn’s test with the Benjamini–Hochberg correction within the *HSPA6*+ comparison. Individual cells were not treated as independent biological replicates. Because several stromal subclusters were screened and the effective groups were small, the resulting statistics are reported as exploratory rather than confirmatory.

We also searched the published Human Endometrial Cell Atlas (HECA) stromal and macrophage differential-expression tables [15] and the supplementary workbook of the 2023 endometriosis genome-wide association study (GWAS) meta-analysis for *FSCN1* and *PCAT1* [20]. These analyses are hypothesis-generating and do not replace the original studies’ cell state annotations or covariate-adjusted models. Reproducible scripts, subject-level summaries, cell counts, and complete statistical outputs are provided in Supplementary File S1.

The schematic figures were prepared with generative AI-assisted drafting (OpenAI ChatGPT Plus 5.5/Codex), followed by scientific review and editing by the authors. The figures are illustrative and do not encode quantitative expression values or measured gradients.

## 3. Working Hypothesis

**Figure 1:**
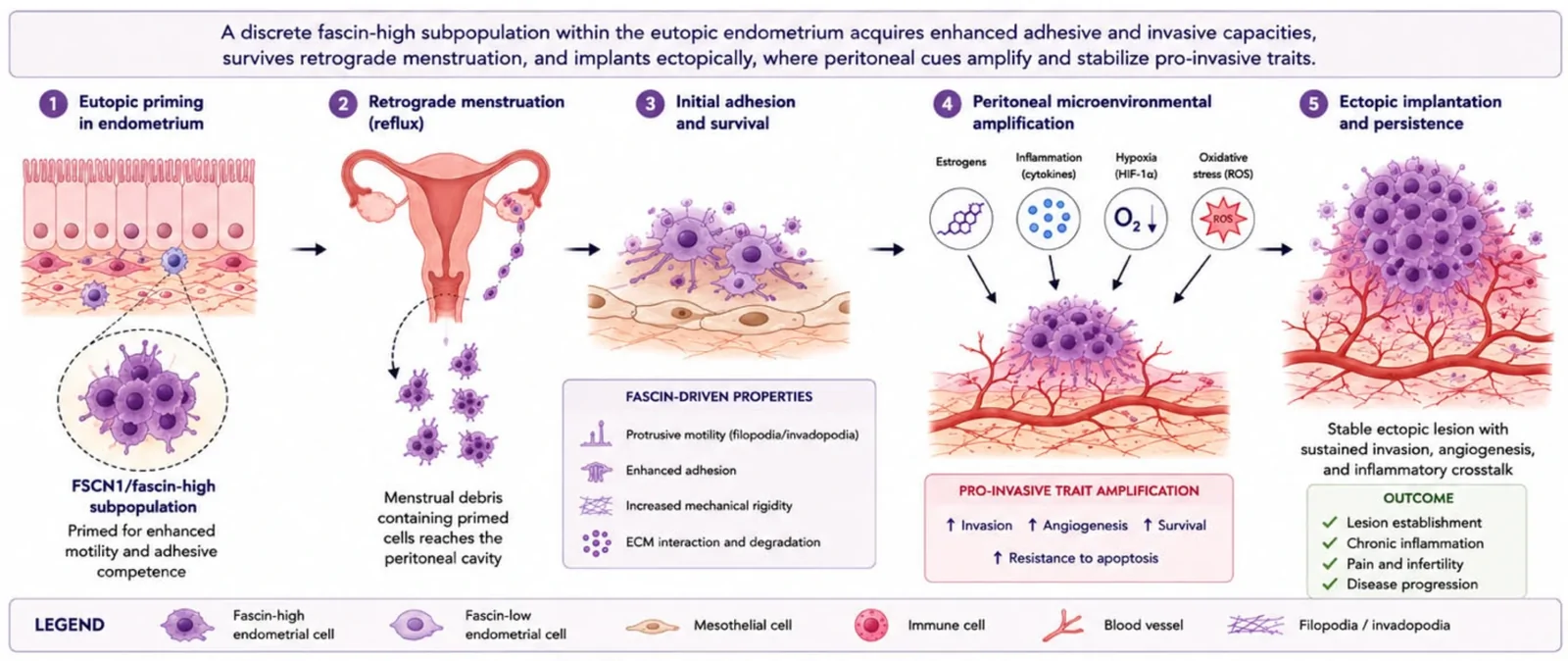
Proposed sequence under Model A (antecedent priming). This conceptual schematic illustrates the hypothesis only; it does not establish a fascin-high eutopic subpopulation, quantitative expression gradient, or measured temporal sequence.

We propose two non-exclusive, testable models. In Model A, a rare fascin-high eutopic cell population has enhanced protrusive and adhesive competence before reflux, increasing the probability of implantation. In Model B, eutopic cells do not show reproducible antecedent enrichment; instead, fascin is induced after adhesion by inflammatory, hypoxic, oxidative, metabolic, hormonal, or biomechanical cues and then reinforces an established invasive phenotype. The current evidence supports fascin as a plausible cytoskeletal effector but does not distinguish these models. Retrograde menstruation provides exposure, whereas cellular composition and the peritoneal niche shape the observed signal and the probability of persistence (Figure 1).

## 4. Endometriosis

### 4.1. Epidemiology, Burden, and Clinical Presentation

Endometriosis affects approximately 10% of women of reproductive age, although prevalence estimates vary with case definition and access to diagnosis [3,21]. Presentations include dysmenorrhoea, deep dyspareunia, chronic pelvic pain, dyschezia, dysuria, infertility, fatigue, and gastrointestinal or urinary symptoms; symptom combinations are neither universal nor pathognomonic, and the disease substantially impairs quality of life [3,4,5,22]. Across settings, intervals of approximately 5–12 years from symptom onset to diagnosis have been reported [3,23].

### 4.2. Clinical Phenotypes and Severity of Disease

The principal pelvic phenotypes are superficial peritoneal disease, ovarian endometrioma, and deep endometriosis. Deep lesions may involve uterosacral ligaments, bowel, bladder, ureters, the pelvic sidewall, and, less commonly, the inferior hypogastric or lumbosacral plexus and other pelvic nerves [8,24,25]. Extrapelvic disease is uncommon but clinically important; thoracic/diaphragmatic and pulmonary sites are the best recognised, whereas central nervous system involvement, including brain lesions, is exceptionally rare [26].

### 4.3. Diagnosis and Unmet Clinical Needs

Diagnosis remains challenging because symptoms overlap with other pain disorders and access to expert imaging varies. The European Society of Human Reproduction and Embryology (ESHRE) and contemporary national guidance no longer require laparoscopy as the routine diagnostic gold standard: pathways combine symptom assessment, examination where appropriate, and transvaginal ultrasound, with specialist ultrasound or pelvic magnetic resonance imaging (MRI) for suspected deep disease, mapping, or inconclusive first-line imaging [1,2,27]. A normal scan does not exclude superficial disease. Laparoscopy is reserved for selected diagnostic or therapeutic indications, and histological confirmation is desirable when tissue is obtained. No blood, endometrial, menstrual effluent, or uterine fluid biomarker is currently recommended as a routine stand-alone diagnostic test [2,28].

Salivary miRNA profiling provides a relevant non-invasive precedent. A 109-miRNA signature (commercialised as Endotest in parts of France and Switzerland) has undergone prospective development and external validation [29,30]. In July 2026, the draft National Institute for Health and Care Excellence (NICE) early-use guidance conditionally recommended Endotest, a saliva-based microRNA test, and EndoSure for a three-year evidence generation period in selected people with suspected endometriosis; neither was proposed as a universal stand-alone replacement for clinical assessment or imaging (available online: https://www.nice.org.uk/guidance/indevelopment/gid-htg10877 (accessed on 28 July 2026)) [31]. This distinction is important when considering any future fascin-centred panel.

### 4.4. Oncomimetic Behaviour and Invasion Logic

Endometriosis is benign but can display invasion, angiogenesis, apoptosis resistance, and migratory plasticity—features sometimes described as oncomimetic [4,5]. The analogy can inform mechanism, but endometriosis is not cancer and oncological findings cannot be transferred without disease-specific validation.

### 4.5. Aetiology and Pathogenesis of Endometriosis

Endometriosis is multifactorial, with hormonal, genetic, immune, developmental, and environmental contributions [3,4,5,20]. The principal, non-mutually exclusive theories are:

1. **Retrograde menstruation theory (Sampson, 1927) [32]:** Viable endometrial cells reflux through the fallopian tubes into the peritoneal cavity during menstruation, where they may adhere, implant, and proliferate on the peritoneal surfaces. This remains the most widely accepted mechanism, although it does not explain why only certain women develop this disease.
2. **Coelomic metaplasia theory:** This theory proposes that the peritoneal mesothelium, derived from the coelomic epithelium, retains the potential to transform into endometrial-like tissue under hormonal or inflammatory stimuli. This mechanism is particularly relevant in rare cases where endometriosis develops in the absence of menstruation or the uterus.
3. **Müllerian remnants theory:** This theory suggests that residual embryonic Müllerian duct cells located outside the uterine cavity may undergo differentiation into endometrial tissue later in life, accounting for deep pelvic or extrapelvic lesions.
4. **Lymphatic or vascular dissemination theory:** This theory proposes that endometrial cells enter lymphatic or blood vessels and metastasise to distant organs, such as the lungs, brain, or surgical scars, explaining rare extrapelvic presentations.
5. **Stem cell theory:** This theory indicates that endometrial or bone marrow-derived progenitor cells with multilineage potential migrate and implant ectopically, giving rise to new endometriotic foci under oestrogenic stimulation.

### 4.6. The Eutopic–Ectopic Continuum Versus Contextual Induction: A Reconciled View

The proposal that intrinsic eutopic abnormalities contribute to lesion formation originates from pathological and molecular comparisons suggesting altered proliferation, adhesion, hormone response, and epigenetic regulation [6,33,34]. However, these findings do not imply that all eutopic cells or all patients share a stable invasive programme.

Riaz et al. found high transcriptomic similarity between eutopic endometrium from women with and without endometriosis, with larger divergence between eutopic and ectopic tissues and no evidence of a universal epithelial–mesenchymal programme [11]. Likewise, a mid-secretory meta-analysis by Vargas et al. found no consistently differentially expressed individual gene after multiple-testing correction, although pathway-level changes in chemotaxis, immunity, locomotion, and receptivity remained [12]. These data permit subtle, phase- or cell state-restricted priming but argue against a globally transformed eutopic endometrium.

Tan et al. analysed more than 122,000 cells from control endometrium, eutopic endometrium, lesions, and organoids, identifying immune tolerance and angiogenesis programmes alongside strong site-specific effects [13]. Importantly, the cohort was hormonally treated, and the published design was not powered to establish a cycle-resolved eutopic *FSCN1* biomarker.

Shih et al. provided a complementary eutopic comparison by profiling endometrial tissues shed into menstrual effluent from controls, symptomatic participants, and surgically confirmed cases [19]. The original study identified five stromal subclusters and reported disease-associated differences in decidualized and inflammatory stromal states. As described below, our exploratory reanalysis suggested that any eutopic *FSCN1* difference may be restricted to the *HSPA6*+ stromal subcluster rather than representing generalised epithelial or whole-tissue activation.

The resulting interpretation is conditional rather than convergent: a permissive or partially primed eutopic substrate may exist in some cell states, while the ectopic niche drives additional vascular, immune, fibrotic, and metabolic adaptation.

Konrad et al. found prominent epithelial–mesenchymal and fibrotic markers in ectopic, but not eutopic, tissue, supporting microenvironmental induction of the established invasive phenotype [14].

The HECA profiled more than 300,000 cells from 63 individuals and highlighted differences in cell abundance, including fibroblast and macrophage compartments, rather than a single disease-specific cell state [15]. Its supplementary endometriosis differential-expression lists for decidual stromal cells and uterine macrophages do not include *FSCN1*. Consequently, higher bulk fascin could reflect altered proportions of endothelial, immune, or stromal cells rather than epithelial induction.

Methodological critiques have likewise warned that tissue type mixing and the use of eutopic endometrium as a surrogate for lesions can distort inference [35]. Any fascin comparison must therefore specify anatomy, cell type, cycle phase, hormonal treatment, and disease phenotype.

Thus, intrinsic predisposition and contextual induction are competing but compatible explanations. Evidence of a cycle-resolved fascin-high eutopic population would favour antecedent priming; absence of such a population, coupled with lesion- or cue-specific induction, would favour fascin as a consequence and amplifier of ectopic adaptation.

A permissive niche may further lower the threshold for adhesion. For example, ectopic milieu succinate can promote stromal cell adhesion through SUCNR1, and early-stage studies describe immune alterations in eutopic endometrium [36,37]. Whether such changes precede implantation, follow occult disease, or reflect treatment remains unresolved; the term ‘pre-endometriotic niche’ is therefore heuristic rather than established.

**Figure 2:**
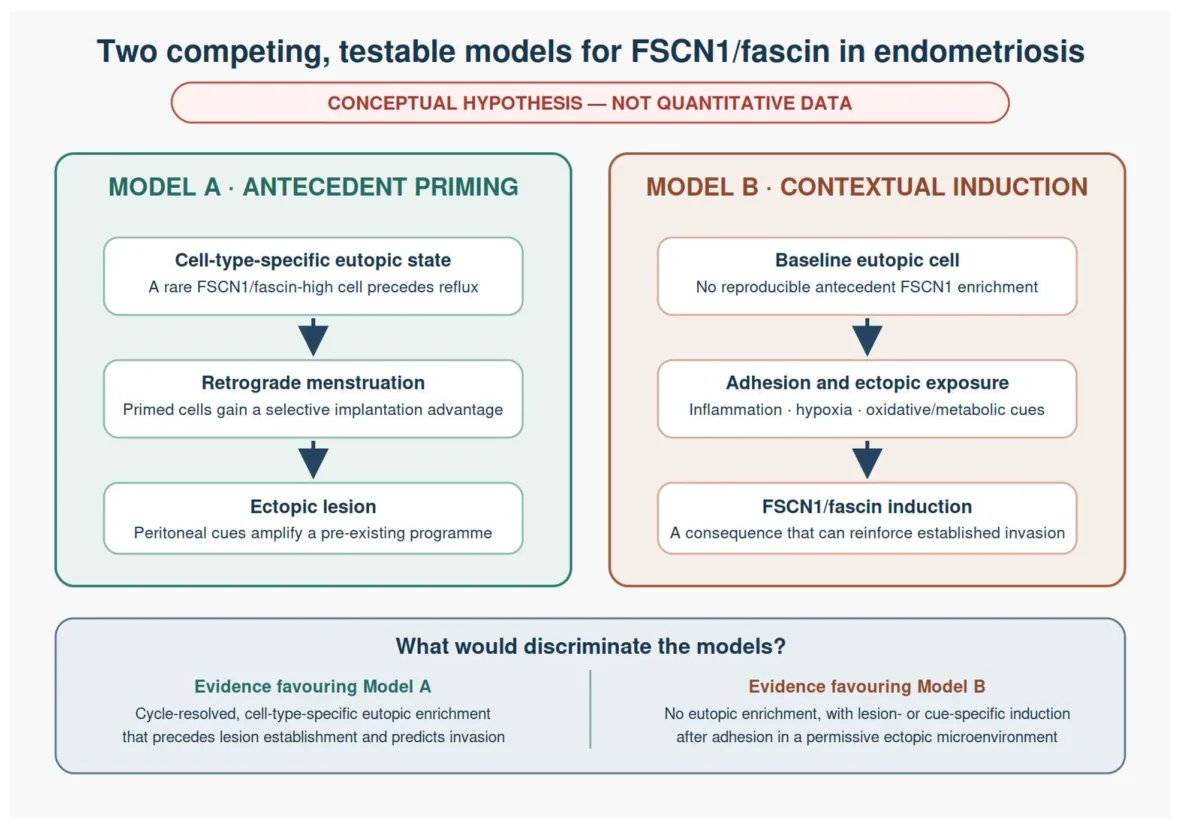
Competing, testable models for *FSCN1*/fascin in endometriosis. Model A proposes antecedent, cell type-specific eutopic priming; Model B proposes induction after ectopic adhesion. The schematic is conceptual and does not represent quantitative expression, a temporal sequence observed in patients, or a validated disease continuum.

Fascin is one candidate cytoskeletal effector within this two-directional framework. Figure 2 explicitly contrasts antecedent priming with contextual induction and identifies observations that could discriminate them; it does not depict measured stages or expression gradients.

## 5. Public-Data Interrogation, Lineage Specificity, and Cell Composition Implications

An exploratory donor-level reanalysis of GSE179640 did not identify statistically significant differences in overall eutopic *FSCN1* expression between controls and patients with endometriosis. In single-cell pseudobulk analyses, the median values in controls and cases were 16.7 and 26.6 counts per million (CPM) across all cells (*p* = 0.48), 1.2 and 5.9 CPM in the marker score-defined epithelial compartment (*p* = 0.37), and 143.4 and 145.5 CPM in the endothelial compartment (*p* = 1.00), respectively. In the independent bulk RNA-sequencing series, the median values were 79.3 and 88.0 CPM in controls and cases, respectively (*p* = 0.53). Although GSE179640 contains multiple anatomical sites, the comparisons above were restricted to control and eutopic endometrium and therefore did not mix eutopic and ectopic tissues.

When anatomical sites within GSE179640 were examined separately, significant *FSCN1* differences were confined to an *ACKR1*-positive endothelial population and a contractile mural cell population. These differences principally separated eutopic endometrium from ectopic lesions, in which *FSCN1* was lower; the evaluable control–eutopic comparison was not significant. Thus, the vascular findings primarily demonstrate anatomical and microenvironmental effects and should not be interpreted as evidence of generalised eutopic *FSCN1* deregulation.

GSE203191 provided an independent analysis of endometrial tissue shed into menstrual effluent. Across the original major-cell annotations, *FSCN1* expression was substantially more prominent in stromal, myeloid, and endothelial cell-like (EC-like) compartments than in epithelial populations. Within the original stromal annotations, *FSCN1* was not a defining marker of the *HSPA6*+ subcluster when this population was compared with the other stromal subclusters. However, within *HSPA6*+ cells, subject-level mean *FSCN1* expression differed nominally among clinical groups (Kruskal–Wallis *p* = 0.0206; η^2^ H ≈ 0.48), with higher values in diagnosed cases than in controls (Dunn’s test, Benjamini–Hochberg-adjusted *p* = 0.016 within this subcluster). The original abundance analysis did not identify significant case–control enrichment of the *HSPA6*+ subcluster. Therefore, the expression observation was not accompanied by a statistically detectable increase in the relative abundance of this population.

This *HSPA6*+-restricted observation should nevertheless be interpreted cautiously. It was obtained from a small post hoc subset, and the global *p* value was not corrected across all stromal subclusters screened. The symptomatic group was not surgically classified and cannot be assumed to represent an intermediate stage of disease. Moreover, the heat-shock and proteostasis programme defining *HSPA6*+ cells could reflect biological stress, preanalytical handling, or dissociation-associated stress. The result therefore identifies a candidate stromal state for validation rather than demonstrating disease progression, causal activation, or increased migratory function.

Taken together, the two datasets argue against uniform or predominantly epithelial eutopic *FSCN1* overexpression. GSE179640 provides no conclusive overall eutopic case–control difference and highlights vascular and anatomical effects, whereas GSE203191 raises the possibility of a lineage-restricted stromal association. Together with the cell-abundance differences reported in the HECA and the absence of *FSCN1* from its published stromal and macrophage differential-expression lists, these findings identify cell composition and cell state as major potential confounders. Apparent differences detected using bulk quantitative polymerase chain reaction (qPCR), unsegmented immunohistochemistry, or whole menstrual effluent assays could reflect variation in vascular, immune, or stromal populations rather than altered expression within endometrial epithelial cells. Lineage-resolved analyses and explicit compositional adjustment are required to distinguish these possibilities.

## 6. Fascin

Fascin, encoded by *FSCN1*, bundles filamentous actin into parallel arrays that support filopodia, dynamic adhesion, directional migration, and invasion [38,39]. It is therefore a functional component of protrusive machinery, not simply a marker.

Physiological expression is prominent in dendritic and other antigen-presenting cells, endothelial cells, and neurones, where fascin contributes to immune cell migration and synapse formation, vascular remodelling, and neuronal morphology [38,40,41]. In normal endometrium, immune and vascular populations participate in cyclical tissue breakdown and repair, but direct cycle-resolved maps of fascin protein in epithelial and stromal compartments remain limited. Across GSE179640 and GSE203191, epithelial *FSCN1* signal was low, whereas non-epithelial expression was prominent; GSE203191 further suggested a preliminary disease-associated difference restricted to the *HSPA6*+ stromal subcluster. This small post hoc observation does not establish the normal physiological baseline, a stable migratory phenotype, or causal involvement in implantation. Establishing fascin expression across proliferative, secretory, and menstrual phases is therefore the first required experiment.

In epithelial cancers, fascin is associated with migration and metastasis, but its mechanical and non-canonical signalling functions are context dependent [39,40,41]. These observations provide an analogy, not proof of an equivalent role in endometriosis.

**Figure 3:**
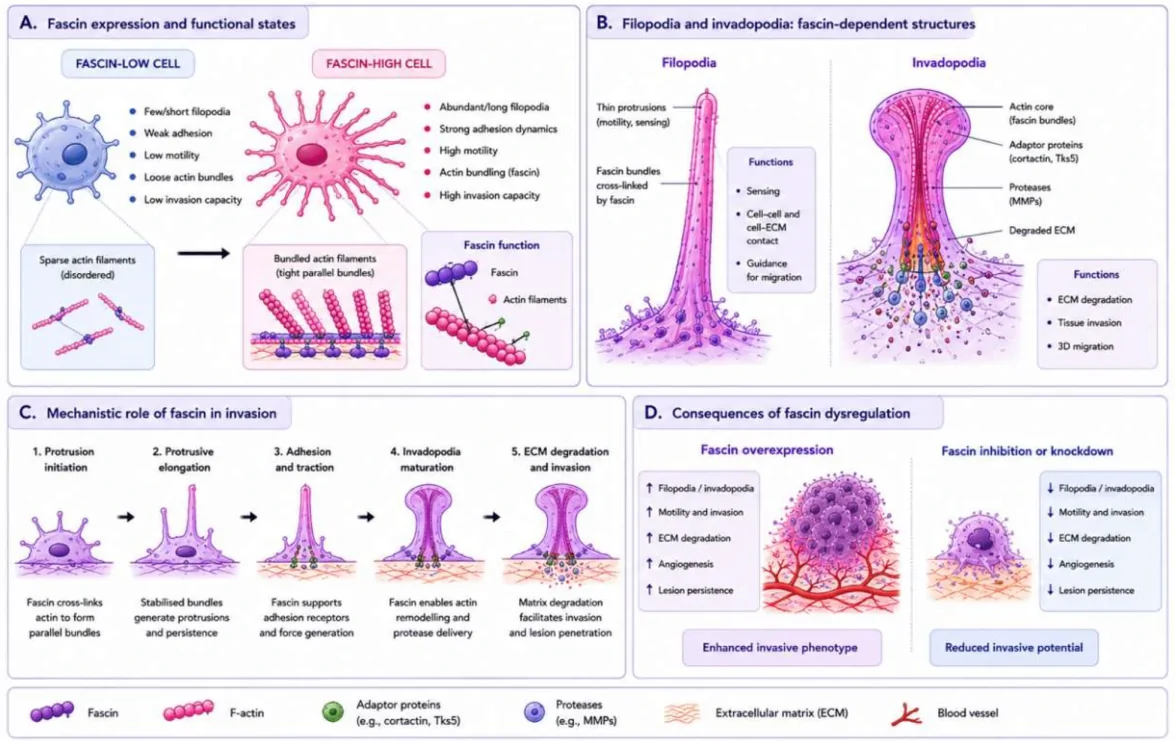
Conceptual overview of fascin biology and its potential contribution to cell invasion. The schematic summarises fascin-mediated actin bundling, filopodial and invadopodial organisation, extracellular matrix remodelling, and the predicted consequences of fascin dysregulation. It does not represent quantitative or endometriosis-specific experimental data.

Ectopic implantation nevertheless requires detachment survival, mesothelial or matrix adhesion, protrusion formation, barrier remodelling, and persistence under inflammatory, oxidative, and hypoxic stress. Fascin is therefore mechanistically plausible as one effector within a broader actin remodelling programme [7,8,18]. The cytoskeletal functions of fascin and its potential contribution to filopodia, invadopodia, extracellular matrix remodelling, and the sequential stages of cell invasion are summarised schematically in Figure 3.

Controlled trophoblast invasion and embryo implantation offer a non-malignant reproductive analogy but do not establish fascin’s function in eutopic endometrium or endometriosis [42].

Ovarian cancer studies provide a peritoneal invasion analogy: genetic or pharmacological fascin inhibition can reduce mesothelial traversal and omental colonisation [43,44]. Because endometriosis is not neoplastic, these findings justify disease-specific experiments but not therapeutic extrapolation.

## 7. Fascin and Endometriosis: Multilevel Support and Its Limits

Endometriosis-specific evidence remains limited. The strongest functional study links autophagy to fascin-dependent invasion: rapamycin reduced proliferation, filopodia, and invasion, while forced fascin expression partially restored invasiveness; diseased specimens showed lower LC3-II and higher p62 and fascin [16]. This rescue result implicates fascin within an autophagy-sensitive programme, but rapamycin is not a fascin-specific intervention and lesion expression does not establish antecedent eutopic activation.

miR-145 provides a second, explicitly non-specific axis. In 12Z cells and primary endometrial stromal cells, miR-145 altered fascin together with pluripotency, adhesion, cytoskeletal, and protease-related targets, including OCT4, SOX2, KLF4, and other effectors [17]. Its phenotypic effects were context dependent and non-linear. Consequently, miR-145 manipulation cannot be interpreted as selective fascin inhibition; target-specific *FSCN1* knockout, re-expression rescue, and parallel quantification of co-regulated targets are required to attribute an invasion phenotype to fascin.

A candidate gene study proposed that *PCAT1* sequesters miR-145 and that rs710886 A>G modulates endometriosis susceptibility and a broader stemness/invasion network [45]. However, neither *FSCN1* nor *PCAT1* is reported among prioritised genes at the 42 genome-wide-significant loci in the substantially larger 2023 GWAS meta-analysis [20]. The candidate association is therefore hypothesis-generating and lacks genome-wide corroboration; it should not be presented as a proven genetic bridge to fascin.

In adenomyosis-derived stromal cells, KDM1A/LSD1 silencing reduced migration and invasion alongside fascin, ezrin, MMP-2, and MMP-9 [46]. This related-disease observation supports a multigene motility module but cannot be treated as direct endometriosis evidence.

Endometriotic stromal cells remodel extracellular matrix and show lesion type-specific differences in migration, contractility, and molecular profiles [47]. The public datasets illustrate why stromal abundance and within-lineage expression must be evaluated separately. In GSE203191, higher *FSCN1* expression was suggested within *HSPA6*+ stromal cells without a statistically detectable increase in the relative abundance of this subcluster. By contrast, GSE179640 highlighted anatomical differences involving vascular-associated populations. A bulk *FSCN1* change could therefore reflect either altered expression within a particular stromal state or changes in the proportions of fibroblasts, macrophages, dendritic cells, mural cells, or endothelium. These alternatives cannot be distinguished without donor-level, lineage-resolved analysis.

**Figure 4:**
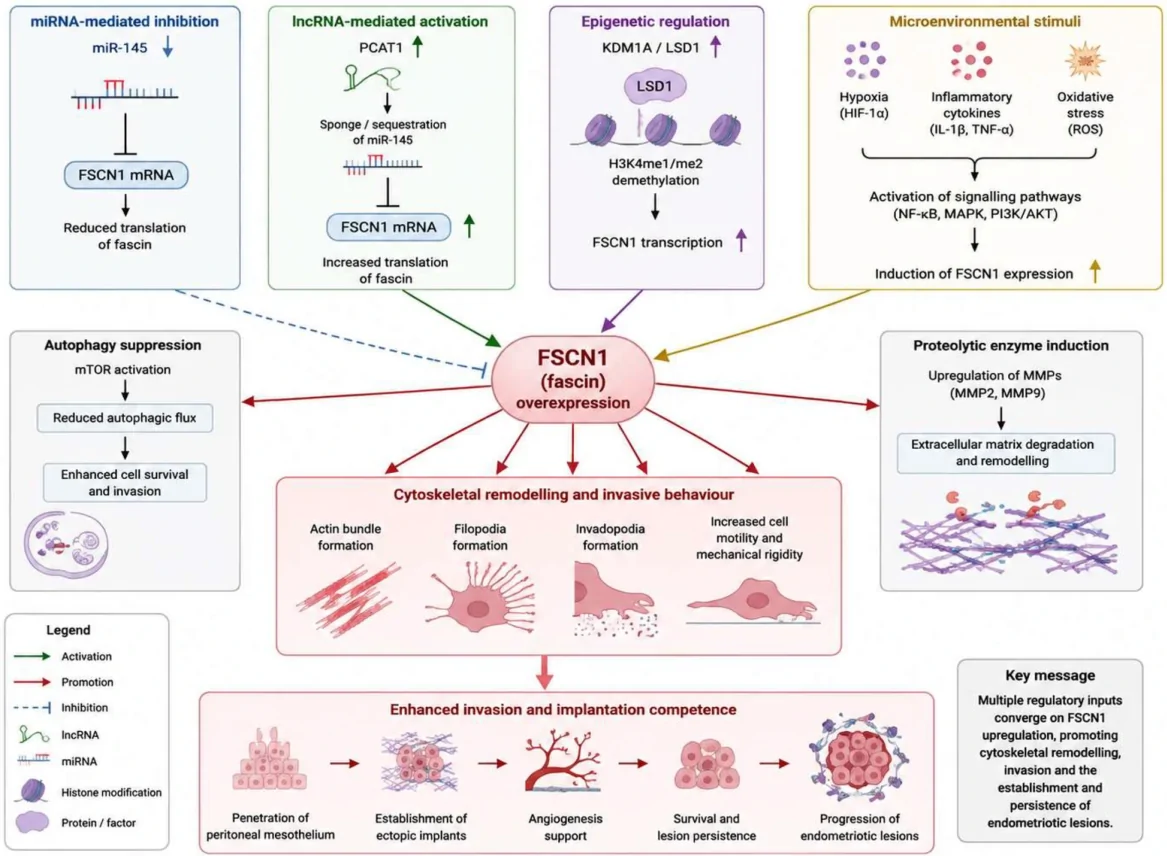
Regulatory network proposed to influence fascin expression and invasion in endometriosis. The network is conceptual; most upstream perturbations are pleiotropic and do not establish FSCN1-specific causality.

These limited lines of evidence place fascin within invasion-relevant networks, but ‘convergence’ would overstate the present literature. Fascin is better described as a candidate integration node whose anatomical, cellular, temporal, and causal specificity remains unresolved (Figure 4).

The decisive issue is whether a reproducible, cycle-resolved fascin-high population exists in eutopic endometrium independently of established lesions. Public datasets do not currently demonstrate generalised eutopic enrichment. However, GSE203191 provides a preliminary signal restricted to a *HSPA6*+ stromal state. This result neither validates a universal antecedent priming model nor supports a purely negative interpretation; instead, it narrows the hypothesis to a potentially rare, lineage-specific state requiring independent spatial and protein-level validation.

Model A predicts a cell type-specific eutopic fascin-high fraction that precedes lesion establishment, carries adhesion/motility signatures, and predicts invasive phenotype. Model B predicts no reproducible eutopic enrichment but inducible fascin after exposure to inflammatory, hypoxic, oxidative, oestrogenic, metabolic, mesothelial, or biomechanical cues. Direct *FSCN1* inhibition should reduce protrusion and invasion in either model if fascin is functionally required; rescue with an inhibition-resistant or re-expressed *FSCN1* construct should restore the phenotype.

The first experiment should map physiological fascin across proliferative, early-, mid-, and late-secretory, and menstrual endometrium in untreated controls using multiplex immunohistochemistry or RNAscope with epithelial (EPCAM/cytokeratin), stromal (VIM/COL1A1), endothelial (CD31/EMCN), leukocyte (CD45), macrophage (CD68), and dendritic cell markers. Within the stromal compartment, *HSPA6* and related heat-shock markers should be quantified to determine whether the exploratory *HSPA6*+ signal is reproduced in intact tissue. Rapid fixation or spatial analysis of minimally manipulated samples will be important to distinguish biological stress states from collection- or dissociation-induced expression. Case–control comparisons should be matched by cycle phase and analysed using donor-level, cell type-specific pseudobulk or segmented protein scores. Flow-sorted compartments, matched eutopic–ectopic samples, and three-dimensional stromal–mesothelial or organoid invasion assays with direct *FSCN1* perturbation should then test causality. Menstrual effluent studies should analyse tissue-enriched and unfractionated preparations separately, record collection and processing delays, quantify epithelial, stromal, leukocyte, and endothelial fractions, and avoid interpreting whole-sample *FSCN1* without compositional adjustment.

A direct validation cohort should include untreated controls, eutopic endometrium from patients with endometriosis, and matched lesions when available, with prospective recording of cycle phase, hormonal treatment, phenotype, anatomy, age, and sampling method. Increased expression confined to lesions would favour contextual induction; cell type-specific eutopic enrichment before or independently of lesion establishment would strengthen antecedent priming.

## 8. Translational Implications

Mechanism-based stratification is attractive, but no single endometrial marker has shown sufficient reproducibility for routine diagnosis, and the current guidelines favour panels and clinically anchored validation over isolated analytes [2,28,48,49,50]. Salivary miRNA validation and the draft early-use guidance illustrate both the feasibility of non-invasive molecular testing and the need for prospective, independent evidence generation [29,30,31].

Fascin should therefore be evaluated only as a candidate component of a composite panel. Its incremental value must be tested beyond symptoms, imaging, cycle phase, treatment, and measured cell composition. Protein-level spatial assays may be more informative than whole-tissue qPCR because they can identify the lineage and subcellular distribution of the signal.

Menstrual effluent is a practically accessible, non-surgical matrix containing exfoliated endometrial, blood, immune, and vascular material. Proteomic studies support analytical feasibility [51,52], but this heterogeneity is also a major confounder: any fascin assay should report collection timing, volume, processing delay, blood dilution, cellular yield, viability, and epithelial/stromal/leukocyte/endothelial proportions. The existing data do not validate fascin as a menstrual effluent biomarker.

Extracellular vesicle miRNA/lncRNA signatures offer another route to interrogate upstream regulatory networks [50,53], but they do not establish that circulating or vesicular fascin is diagnostically useful.

Therapeutic interpretation requires greater caution than biomarker exploration. Fascin acts in normal dendritic cell migration and immune synapse organisation, endothelial remodelling and repair, and neuronal morphology [38,40,41]. Systemic inhibition could therefore affect immune surveillance, vascular or wound repair, and neural function; oncology safety data cannot define the risk–benefit balance for a chronic benign disease. Direct fascin targeting in endometriosis remains preclinical [16,18,45].

Repurposed or investigational agents with anti-fascin activity, including imipramine, raltegravir, and NP-G2-044, may be useful as mechanistic probes [54,55,56], but off-target effects and clinically relevant exposure must be separated from *FSCN1*-specific action. If efficacy is confirmed, risk mitigation strategies could include short-course rather than continuous exposure, intraperitoneal biodegradable depots or hydrogels, lesion-targeted nanoparticles or ligand-directed carriers, and local administration at surgery. These approaches remain experimental, may not reach multifocal or extrapelvic disease, and require biodistribution, fertility, immune, vascular repair, neurotoxicity, and wound healing assessment before clinical translation.

## 9. Limitations and Alternative Interpretations

Endometriosis varies by phenotype, lesion site, cycle phase, hormonal treatment, sampling method, and cell composition. Elevated fascin may be causal, permissive, or consequential: inflammation, hypoxia, oxidative stress, or biomechanical cues could induce it after implantation, while enrichment of endothelial, dendritic, macrophage, mural, or fibroblast populations could increase bulk measurements without a within-lineage change. Although our GSE179640 case–control analysis excluded ectopic tissues, the cohort contained only 3 control and 9 eutopic endometriosis donors; most participants were hormonally treated, and the broad marker score annotation cannot resolve rare or transient cell states.

GSE203191 provides a more anatomically appropriate eutopic comparison but derives from menstrual effluent rather than conventional endometrial biopsy. Its source study included tissue-enriched and unfractionated preparations, and the *HSPA6*+ analysis was restricted to a small post hoc subset. The nominal global association was not corrected across all stromal subclusters screened. The symptomatic group lacked surgical classification and cannot be interpreted as an intermediate stage of disease. In addition, the heat-shock programme defining *HSPA6*+ cells may partly reflect collection, transport, fixation, or dissociation stress. Consequently, the observed association cannot establish temporal progression, causality, increased migratory capacity, or independence from preanalytical effects.

Systematic, lineage-resolved protein studies in paired control eutopic, case eutopic, and ectopic tissues are lacking. A reproducible *HSPA6*+-restricted signal in rapidly preserved intact tissue would support a cell state-specific eutopic association, whereas the absence of such validation would favour a technical or context-dependent explanation. Neither possibility establishes that fascin activation precedes lesion formation.

Rapamycin and miR-145 perturb multiple pathways, and miR-145 regulates numerous pluripotency, adhesion, protease, and cytoskeletal targets. Causal attribution therefore requires direct *FSCN1* knockout or knockdown, rescue, and orthogonal pharmacological confirmation.

Parts of the argument derive from cell lines, small cohorts, adenomyosis, or cancer analogies. The candidate *PCAT1* association also lacks corroboration among genome-wide-significant endometriosis loci [20]. These sources generate hypotheses but do not establish clinical utility.

Finally, fascin is unlikely to act alone. Even a biologically valid signal would require preanalytical standardisation and demonstration of incremental value in a multi-marker model, not stand-alone deployment.

## 10. Conclusions

Fascin is a mechanistically plausible actin bundling effector in endometriosis, but the current evidence does not establish that generalised eutopic *FSCN1* activation precedes implantation. Two non-exclusive models remain viable: a rare, cycle- and cell state-specific eutopic population that may increase implantation competence, and contextual induction after ectopic adhesion that reinforces established invasion. The available functional studies support a role within broader autophagy- and miR-145-sensitive networks, not a solitary or validated disease driver [16,17].

The public-data screen produced a heterogeneous but informative picture. GSE179640 did not identify a conclusive overall donor-level difference between control and endometriosis eutopic endometrium and showed that vascular *FSCN1* differences were strongly influenced by anatomical site. GSE203191 suggested higher *FSCN1* expression within a *HSPA6*+ stromal subcluster in diagnosed cases, without a statistically detectable increase in the abundance of that subcluster, but this small post hoc observation requires correction across the full analytical testing family and independent validation. Neither dataset provided convincing evidence of generalised epithelial upregulation. The absence of *FSCN1* from the reported HECA stromal/macrophage differential-expression lists and the lack of genome-wide prioritisation of *FSCN1* or *PCAT1* [13,15,20] further constrain the hypothesis.

The next step is a cycle-resolved physiological map, followed by lineage-resolved validation in rapidly preserved, matched control eutopic, case eutopic, and ectopic tissues. Particular attention should be paid to *HSPA6*+ stromal cells, preanalytical stress, cell composition, and direct *FSCN1* perturbation in functional invasion models. Until such evidence is available, fascin should be treated as a candidate component of multi-marker research and a preclinical mechanistic target. Systemic inhibition is not justified, and any future therapeutic development should prioritise local or lesion-directed exposure and formal immune, vascular, neural, reproductive, and wound healing safety assessment.

## Data Availability

Public transcriptomic data are available from the NCBI Gene Expression Omnibus under the accessions GSE179640 and GSE203191. The HECA differential-expression tables accompany Marečková et al. [15], and the GWAS summary resources accompany Rahmioglu et al. [20]. The analysis scripts, donor- and subject-level-derived summaries, cell count thresholds, and complete exploratory statistical outputs are provided in Supplementary File S1.

## Supplementary Materials

The following supporting information can be downloaded at: https://www.mdpi.com/article/10.3390/ijms27167234/s1.
